# The activity in the contralateral primary motor cortex, dorsal premotor and supplementary motor area is modulated by performance gains

**DOI:** 10.3389/fnhum.2014.00201

**Published:** 2014-04-16

**Authors:** Ronen Sosnik, Tamar Flash, Anna Sterkin, Bjoern Hauptmann, Avi Karni

**Affiliations:** ^1^Department of Neurobiology, Brain Research, Weizmann Institute of ScienceRehovot, Israel; ^2^Department of Computer Science and Applied Mathematics, Weizmann Institute of ScienceRehovot, Israel; ^3^Faculty of Medicine, Goldschleger Eye Research Institute, Tel Aviv UniversityTel Hashomer, Israel; ^4^Department of Neurology, Segeberger KlinikenBad Segeberg, Germany; ^5^Department of Therapeutic Sciences, MSH Medical School HamburgHamburg, Germany; ^6^Faculty of Education, Department of Learning Disabilities, The Brain Behavior Research Center, University of HaifaHaifa, Israel

**Keywords:** motor learning, motion smoothness, fMRI, movement sequence, co-articulation

## Abstract

There is growing experimental evidence that the engagement of different brain areas in a given motor task may change with practice, although the specific brain activity patterns underlying different stages of learning, as defined by kinematic or dynamic performance indices, are not well understood. Here we studied the change in activation in motor areas during practice on sequences of handwriting-like trajectories, connecting four target points on a digitizing table “as rapidly and as accurately as possible” while lying inside an fMRI scanner. Analysis of the subjects' pooled kinematic and imaging data, acquired at the beginning, middle, and end of the training period, revealed no correlation between the amount of activation in the contralateral M1, PM (dorsal and ventral), supplementary motor area (SMA), preSMA, and Posterior Parietal Cortex (PPC) and the amount of practice *per-se*. Single trial analysis has revealed that the correlation between the amount of activation in the contralateral M1 and trial mean velocity was partially modulated by performance gains related effects, such as increased hand motion smoothness. Furthermore, it was found that the amount of activation in the contralateral preSMA increased when subjects shifted from generating straight point-to-point trajectories to their spatiotemporal concatenation into a smooth, curved trajectory. Altogether, our results indicate that the amount of activation in the contralateral M1, PMd, and preSMA during the learning of movement sequences is correlated with performance gains and that high level motion features (e.g., motion smoothness) may modulate, or even mask correlations between activity changes and low-level motion attributes (e.g., trial mean velocity).

## Introduction

Numerous electrophysiological and neuroimaging studies have attempted to examine the role that different cortical and sub-cortical motor areas subserve in the planning and execution of planar hand movements. The amount of activation in different motor areas was suggested to correlate with various temporal and spatial properties of arm movement, e.g., hand velocity (Lewis et al., 2003; Wang et al., 2007; Casabona et al., 2010; Ifft et al., 2011), force (Kalaska and Crammond, 1992; Dettmers et al., 1995; Sehm et al., 2010), muscle activation (Todorov, 2000a, 2002b; Scott et al., 2001), hand position (Georgopoulos et al., 1982; Kettner et al., 1988; Wang et al., 2007), and movement direction (Georgopoulos et al., 1982; Schwartz et al., 1988; Naselaris et al., 2006; Polyakov et al., 2009). It has been shown that the activity of single cells is correlated with multiple motion variables, the same movement variable is represented in multiple areas and representations within a structure are labile (Alexander and Crutcher, 1990; Ebner and Fu, 1997; Turner et al., 1998; Moran and Schwartz, 1999a,b; Eisenberg et al., 2010; Mollazadeh et al., 2011).

A prolonged training on a new motor task is usually followed by enhanced performance, measured by a reduction in reaction time and the number of errors and/or by changes in movement synergy and kinematics (Karni, 1996; Doyon et al., 1997; Shadmehr and Holcomb, 1997; Sosnik et al., 2004, 2007). There is no consensus regarding the neural substrates mediating the incremental acquisition of skilled motor behavior and much less is known with regard to the nature of the dynamic neural changes that occur in the motor system during the different phases of learning. While some works have reported an increase in the magnitude or extent of activation in particular brain areas as an effect of practice, possibly due to increased neural recruitment (Grafton et al., 1995; Karni et al., 1995; Ungerleider et al., 2002; Penhune and Doyon, 2005; Duff et al., 2007), others have reported no change or a decrease in the activation in these brain areas with increased amount of practice, possibly due to the development of representations that produce the same behavior with higher neural efficiency (Buckner et al., 1995; Toni et al., 1998; Ungerleider et al., 2002; Poldrack et al., 2005; Ma et al., 2010; Gobel et al., 2011; Diedrichsen and Wiestler, 2013). Moreover, in some reports, a shift in the activity to other brain areas along with the acquisition of skill was observed (Raichle et al., 1994; Krebs et al., 1998; Poldrack et al., 1998; Olesen et al., 2004; Naumer et al., 2009; Albouy et al., 2012). The contradicting findings may partially result from the use of different experimental tasks or having the subjects practice for different, usually limited periods of time. A little number of imaging studies have investigated the changes in motor representation that occur in the brain over the entire course of motor learning (Karni et al., 1995, 1998; Toni et al., 1998; Kelly and Garavan, 2005; Diedrichsen and Wiestler, 2013; Penhune, 2013). Moreover, inference about the neural correlates of skill acquisition was solely based on the change in the amount of activation as a function of the amount of practice. Given that different subjects may attain different levels of motor proficiency (performance gains, e.g., velocity, accuracy, smoothness) throughout the training period (Sosnik et al., 2004, 2007; Bruce et al., 2010), correlating the subjects' pooled amount of activation with the amount of practice *per se* at different time points throughout the training period may result in inconsistent results.

We previously showed that extensive training on a sequence of planar hand trajectories passing through several targets resulted in the co-articulation of movement components (the spatial and temporal overlap of the adjacent units) and in the formation of new movement elements (primitives) (Sosnik et al., 2004, 2007). Reduction in movement duration was accompanied by the gradual replacing of straight trajectories by longer curved ones, the latter affording the maximization of movement smoothness (“global motion planning”). In the current study we aimed at unraveling whether the engagement of the different motor areas in the acquisition of the new motor skill is correlated solely with the stage and amount of practice (number of repetitions) common to all subjects or is only/also dependent on individual levels of motor proficiency. We further aimed at studying the correlation between trial by trial changes in activation and the corresponding changes in low (velocity) and high (smoothness) motion features.

## Materials and methods

### Ethics statement

The study was approved by the WIS Ethics Committee. Informed consent was obtained from all subjects.

### Behavioral task

#### Training outside the MRI scanner

**Figure 1:**
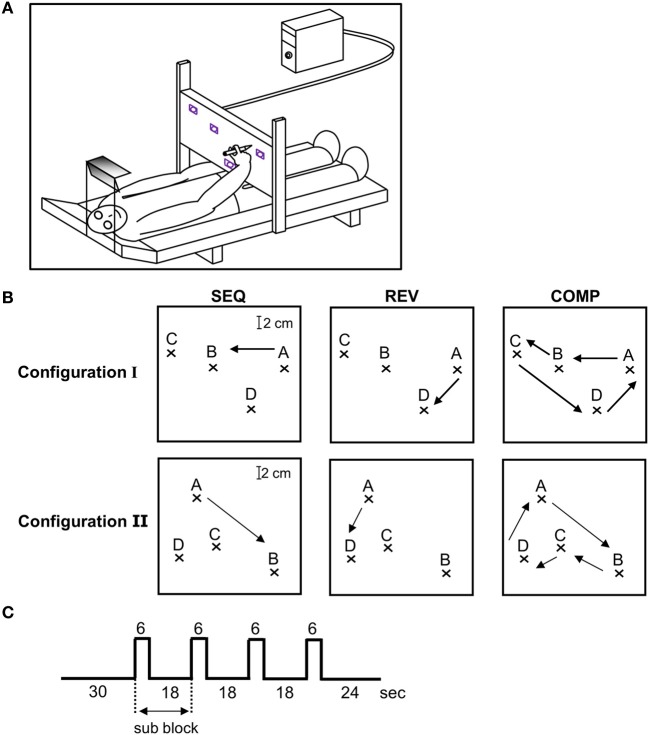
**Experimental design. (A)** Experimental set-up. The recording system outside the fMRI scanner was composed of a bed surrounded by an adjustable metal scaffold. A digitizing tablet was mounted on the scaffold above the subject's hip level in the horizontal plane crossing the hip at a convenient distance to reach the tablet with a pen. To minimize shoulder movements, prevent any head movements and guarantee an fMRI-like head position, subjects looked through a double mirror system at the workspace of the tablet. The field of view, head position, and body position as well as drawing distance during the behavioral training were comparable to the situation in functional magnetic resonance imaging sessions. **(B)** The three training conditions—SEQ, REV, and COMP depicted on target configuration I (three upper plots) and II (three lower plots). The arrow depicts movement direction. **(C)** A single event design was used in which a block was composed of a baseline phase (30 s) followed by four alternating movements (two scans of 3 s each) and resting periods (six scans of 3 s each).

Five right handed subjects (three males and two females aged 25–35 years) participated in the study. The only criterion used to determine which hand is dominant was the hand they reported using for writing. The subjects were trained for 9 days (sessions), spaced 1–3 days apart. Each training session lasted about an hour. A training session was composed of 10–15 training blocks each consisting of 20 trials. There was a two-second rest between two consecutive trials and 1-minute rest between two consecutive blocks. Subjects were placed in a supine posture on a bed (simulating posture in the MRI scanner) and looked at the workspace (digitizing table) through a double mirror system (Figure 1A). The field of view enabled the subjects a clear view of the whole work area without moving or lifting up the head. The digitizing table (WACOM INTUOS, 616 × 446 × 37 mm, resolution 100 ppi, max. data rate 200 pps, accuracy ± 0.25 mm) was mounted on the scaffold above the subject's hip level in the vertical plane at a convenient distance for the subject to reach the table with a pen (cordless, 13 g weight). A convenient drawing distance was further guaranteed by adjusting the height of the digitizing table for each subject individually. The subjects were instructed to avoid lifting the tip of the pen from the work space surface. To minimize shoulder movements, prevent any head movements and guarantee and fMRI-like head position, the head was restrained by a plastic head holder (frame) and rubber foam pads. In order to minimize friction, targets (black crosses of 10 × 10 mm) were printed on commercial transparencies that were attached to the surface of the digitizing table. Digital data were streamed to computer disk for off-line analysis.

The task consisted of a sequence of point-to-point movements where subjects were asked to connect four target points (ABCDA) “as rapidly and as accurately as possible” with their dominant hand upon the hearing of a tone (SEQ condition). Subjects were divided into two groups which practiced different target configurations: I and II. Three subjects (#1, #2, and #3) trained on target configuration I, which had two pairs of highly spatially co-aligned segments (*AB*;*BC* and *CD*;*DA*) and two subjects (#4 and #5) trained on target configuration II, which had just one pair of highly spatially co-aligned segments (*BC*;*CD*). The motivation for selecting two different target configuration was to dissociate between changes in brain activation due to a decrease in the number of generated segments throughout training (four segments to two segments after full co-articulation in target configuration I as opposed to four segments to three segments after full co-articulation in target configuration II) to changes occurring due to the prolonged practice period, irrelevant of task configuration.

#### Training inside the MRI scanner

The subjects were tested inside the MRI scanner at three different time points throughout training: at the beginning (Day 1—denoted as “scanning day 1”), middle (Day 6—denoted as “scanning day 2”) and end of the experiment (Day 9—denoted as “scanning day 3”). The subjects were given the same instructions as were given outside the MRI scanner (“move as rapidly and as accurately as possible”). For each target configuration (I and II), functional brain images were acquired while subjects performed the trained sequence—SEQ (ABCDA, Figure 1B top and bottom left panel), the trained sequence but in a reverse direction—REV (ADCBA, Figure 1B top and bottom middle panel), and the four sequence components wherein the subjects had to fully stop after each sequence component—COMP (AB-BC-CD-DA, Figure 1B top and bottom right panel). Conditions REV and COMP were performed only inside the magnet as opposed to the SEQ condition that was heavily trained outside the magnet. The purpose for including the REV condition was to test whether the changes in brain activation found in the SEQ condition, are related to the amount of practice (which was smaller for REV condition) or to the performance gains by the end of the training sessions (which was almost similar to SEQ). The purpose for including the COMP condition was to further test whether the changes in brain activation are related to the amount of practice (which was similar to REV condition) or to the performance gains (which was lower than in REV condition). The results obtained outside and inside the MRI scanner for the SEQ condition were not qualitatively or quantitatively different (Hauptmann et al., 2009).

Each performance session inside the magnet consisted of 12 blocks in which only one of the three conditions (SEQ, REV, or COMP) were performed (the performing conditions were presented in a randomized order and announced by headphones). Each performance block consisted of four sub-blocks. In each sub-block the imaging data were acquired for 6 s while performing (active images), and for 18 s while resting (rest images) with the eyes looking at the target screen. The subjects repeated the full sequence twice in each sub-block, thus, each movement was repeated for eight times in each performing block for a total of 96 movements in a performing session. Figure 1C depicts the experimental design.

#### Drawing system and movement recording system inside the MRI scanner

The experimental setup is described in detail in Hauptmann et al. (2009). Briefly, the functional MR imaging compatible movement recording system consists of a translucent semicircular plastic board (width 415 mm, length 430 mm), a stylus (i.e., a plastic pen with a fiber-optic core, connected through fiber optics to a halogen light power source) of comparable size and weight to the one provided with the commercial digitizer tablet, a commercially available CCD camera (Pulnix TM-300, 1/2″ CCD sensor, nominal resolution 752H × 582V, video format analog CCIR), a video monitor and a PC with a proprietary video-grabbing card for light detection (grabber card). The grabber card collects only data points whose intensity exceeds a given threshold. While scanning, the behavioral data was sampled at 100 Hz.

In the MRI scanner, the subject wore either prism glasses or used a mirror device that guaranteed the same field-of-view (FOV) and a comparable work space as in the behavioral experiments. Headphones were used for the auditory signal input. Similarly to the behavioral set-up, the head was restrained by a plastic head holder (frame) and rubber foam pads on both sides. After positioning the subject in a supine position inside the MRI scanner bore, the workspace pad was clamped to the inner wall of the bore within a convenient distance for drawing (hand-writing like) movements, in similarity to the set-up for the behavioral control experiment. From that stage on, the subject was instructed to remain “as still as possible.”

#### fMRI data acquisition and preprocessing

We used a 2T MRI Elscint scanner system, equipped with echo planar imaging (EPI) capabilities using the standard head coil for radio-frequency (RF) transmission and signal reception. Using a mid-sagittal scout image, 12 axial slice positions (no gap) were oriented parallel to the bi-commissural plane with the uppermost slice aligned 5 mm below the vertex, thus approximately covering most of the brain (specifically, covering the primary motor cortex and the lateral and medial pre-motor areas). Another six slices (1 mm inter-slice gap) covering the cerebellum were acquired. T1-weightened images showing the anatomical structure and functional images measuring the brain activation were acquired [repetition time (TR) = 11.4 ms, echo time (TE) = 4.4 ms, 15° flip angle, field of view = 256 × 256 mm^2^, matrix size = 200 × 256, 128 sagittal slices with 1.33 mm thickness] to determine the anatomical landmarks.

In each experiment a time series of 132 scans was acquired. In each scan, a set of 44 axial T2^*^-weighted gradient-echo echo-planar images (TR = 3 s, TE = 48 ms, FOV = 220 ^*^ 220 mm^2^, matrix size = 64 ^*^ 64, voxel size = 3.43 ^*^ 3.43 ^*^ 5 mm^3^, slice thickness 5 mm) were collected. We used a single event design in which a baseline phase (30 s) was followed by four alternating movements [an alternating movement being ABCDA (SEQ), ADCBA (REV), or AB-BC-CD-DA (COMP)] of two scans (3 s each) and resting (six scans of 3 s each) periods. The imaging data were acquired for 6 s while performing (active images), and for 18 s while resting (rest images) with the eyes looking at the target screen. Three seconds before the end of the respective resting conditions subjects were instructed to lift the arm upon a computer generated acoustic “get ready” signal and each movement sequence was triggered by an acoustic “go” signal. Timing and accuracy of each movement sequence was visually controlled by a video monitor outside the scanning room. At the end of each imaging session the movement recording system was calibrated again.

All images were analyzed using Statistical Parametric Mapping 2008 Software (Wellcome Department of Cognitive Neurology, London, UK, http://www.fil.ion.ucl.ac.uk/spm/). Before applying statistical analysis, several preprocessing procedures were performed. Initially, origin coordinates were adjusted to the anterior commissure (AC) and the first four data sets of each time series and the last scan of each resting condition (containing the arm lifting movement) were discarded. The remaining EPI volumes were corrected for head movements within and across runs using a rigid-body rotation and translation algorithm (spatial realignment using sinc interpolation) (Friston et al., 1996). The realigned images were also corrected for differences in acquisition time between slices (using sinc interpolation) resulting in each time series having the values that would have been obtained had the slices been acquired at the same time. Finally, the images were spatially normalized to the standard space of the Montreal Neurological Institute brain (MNI brain) and convolved with an isotropic Gaussian kernel [full-width half maximum (FWHM) = 8 mm] to increase signal-to-noise ratio. A high-pass filter (0.015 Hz) was used to remove low-frequency drifts and fluctuations of the signal (Friston et al., 1996), and proportional scaling was applied to remove global changes in the signal.

#### fMRI data analysis

We compared the mean image during movement with the mean image during rest for each sub-block, adjusting for the hemodynamic lag (i.e., the detection of voxels exceeding threshold was always done for movement (SEQ, REV, or COMP) vs. rest period). We used a normal general linear model (GLM) approach with boxcar regressors, convolved with the HRF and the baseline, adjusting for the hemodynamic lag. In order to account for task correlated head movements, the six movement parameters computed in the realignment procedure were added to the design matrix as covariates (regressors). Voxels with a significant change of signal intensity [*p* < 0.05, Student two-tailed *t*-test corrected for FDR (false discovery rate)] compared with the rest period were considered as active.

In order to relate to studies, which have reported changes in the extent of activation in the different motor ROIs (regions of interest, see next section) following practice, we aimed at getting a voxel count measure rather than extracting percent signal change in a hot-spot region, as is usually performed. To that end, we exported the ROIs and the activity maps (of every subject in every condition in each scanning day) to the MRIcro software (University of Nottingham, Nottingham NG7 2RD, UK, http://www.cabiatl.com/mricro/mricro/mricro.html) wherein we used a simple existing routine in order to find the number of voxels activated in the different brain areas of each subject (i.e., number of voxels exceeding threshold).

#### ROIs construction

We aimed at monitoring the amount of activation throughout training in the primary motor area (M1) which is believed to represent low level attributes of the movement such as hand position and velocity, the secondary motor areas which include the Premotor [PM, both dorsal (PMd), and ventral (PMv)], the supplementary motor area (SMA, both SMA proper and pre-SMA), which are believed to represent higher aspects of the motion such as the concatenation of simple movements into more complex structures and the Posterior Parietal Cortex (PPC), which is believed to extract information on visual motion for perception as well as for the guidance of movements.

**Figure 2:**
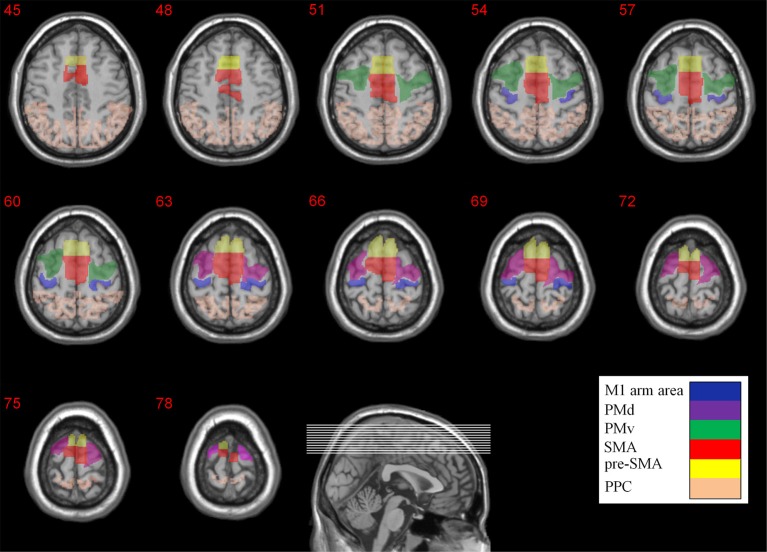
**Anatomical locations of the different ROIs**. The M1 arm area, PMd, PMv, SMA, pre-SMA, and PPC are color coded. The Z coordinates are depicted above each transverse slice.

In order to quantify the extent of activity in the different brain areas we firstly imported the anatomical location of M1, PM (PMd and PMv), SMA (SMA proper and pre-SMA), and PPC from the WFU_PickAtlas toolbox (Maldjian et al., 2003, 2004) and later modified them manually for each subject individually using the ROI drawing tool available in the MRIcro software. For the upper limb region in M1 (including the fingers, hand, wrist, and elbow) the ROI specification was based on the cortical maps and the coordinates reported in Alkadhi et al. work (Alkadhi et al., 2002a,b). Subdivisions of Premotor cortex (PM) were recognized on the basis of their relative locations within area 6. The location of the border between dorsal (PMd) and ventral (PMv) zones was determined based on the gyral branch that divides the inferior Precentral sulcus from the superior Precentral sulcus (Tomassini et al., 2007). As there is no local anatomical landmark that differentiates SMA proper and pre-SMA in the human brain, the vertical line from the anterior commissure (VCA line) was used as the best approximation (Zilles et al., 1996). The anatomical locations of the different ROIs are depicted in Figure 2.

#### Assessing performance gains

Two motion features were assessed: trial mean velocity (low level performance gains) and motion smoothness (high level performance gains).

#### Assessing trial mean velocity

The trial mean velocity was assessed by dividing the area under a trial tangential velocity curve by the trial total movement duration.

#### Assessing motion smoothness

Motion smoothness was assessed by the fit of the trajectories predicted by the minimum-jerk model to the generated trajectories. The minimum jerk model assumes that given a start position, end-point position and the position of one or more via-points (the path locations at which a local minimum velocity is attained, corresponding to the point of local maximum curvature), the system preplans an entire hand trajectory that passes through all these points with the smoothest possible (minimum jerk) trajectory. The objective cost function (cost) to be minimized is the square of the magnitude of the jerk (rate of change of acceleration) of the hand integrated over the entire movement (Flash and Hogan, 1985; Sosnik et al., 2004, 2007).

The fit to the “global planning” model is assessed using an index that incorporates both the normalized velocity error and the normalized path error. Thus, the fit index for one pair of movement segments is:

$$Fit\_index=1-\frac{Normalized\_velocity\_error+Normalized\_path\_error}{2}$$

Wherein:

$$\begin{array}{l} Normalized\_velocity\_error=\frac{\left|Vel_{model}-Vel_{data}\right|}{\left|Vel_{model}-Vel_{data}\right|+Vel\_curve\_length}\\ Normalized\_path\_error=\frac{\left|Path_{model}-Path_{data}\right|}{\left|Path_{model}-Path_{data}\right|+Path\_curve\_length} \end{array}$$

**Figure 3:**
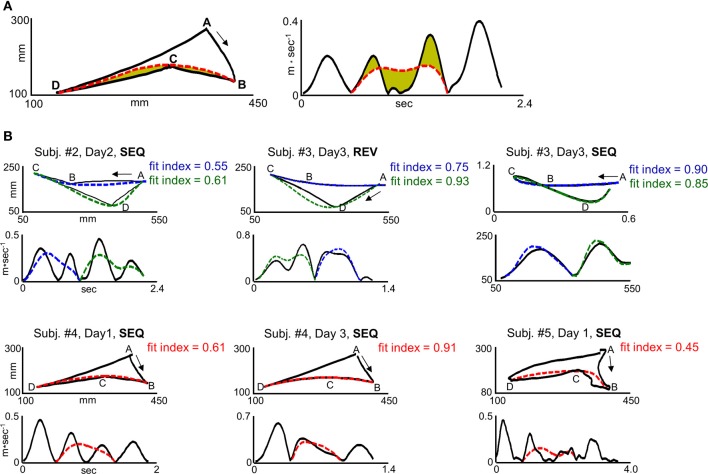
**“Global planning” model fit indices for different trajectory shapes. (A)** Computation of the fit index for segment *BCD* in target configuration I for a representative SEQ trial in scanning day 1. I. The left plot and right plot depict path and velocity profile, respectively. The solid black curve and dashed red curve depict generated and predicted trajectory, respectively. The green colored area in the left and right plot depicts path and velocity error, respectively. The path error was normalized by the path error + length of the path error curve. The velocity error was normalized by the velocity error + length of the velocity error curve. **(B)** Fit indices computed for different trajectory shapes and their corresponding velocity profiles. The three upper panels and lower panels depict trials generated in target configuration I and II, respectively. Upper plots, paths. Lower plots, velocity profiles. The solid lines denote the generated trajectories. The blue and green dashed lines denote the predicted trajectory for movement elements *ABC* and *CDA*, respectively, in target configuration I. The red dashed line denotes the predicted trajectory for movement element *BCD* in target configuration II. Jerky paths, straight paths, and curved paths result in weak fit index (fit index < 0.5), moderate fit index (0.5 = fit index = 0.75) and a strong fit index (fit index > 0.75), respectively.

The term |*Vel_model_* − *Vel_data_*| denotes the velocity error—the area that lies between the generated velocity curve and the predicted velocity curve (Figure 3A, right plot, shaded green area) and the term |*Path_model_* − *Path_data_*| denotes the path error—the area that lies between the generated path and the predicted path (Figure 3A, left plot, shaded green area). The velocity curve length and path curve length are the closed curves bounding the velocity error and the path error, respectively (Figure 3A, left plot dashed curve and right plot dashed curve, respectively). Since, as was shown previously (Sosnik et al., 2004, 2007), the transition from generating two straight point-to-point trajectories to generating a smooth, curved trajectory was accompanied with concurrent reduction in the velocity error and path error, we assigned the same weight to the two errors and calculated a simple mean error. Finally, in order to attain an index which is positively correlated with motion smoothness, the mean error was subtracted from 1.

### Target configuration I

#### SEQ and REV conditions

With the assumption that neighboring segments are co-planned, the minimum jerk model was applied to two movement elements—*ABC* and *CDA*, assuming a single via-point for each movement element (the “global planning” model; Sosnik et al., 2004, 2007).

It was found that connecting targets *ABC* or *CDA* with two straight paths (*AB* and *BC* for movement element *ABC*, *CD* and *DA* for movement element *CDA*) results in a fit index of 0.3–0.5, wherein a fit index of 0.3 corresponds to the generation of a non-maximally smooth straight point-to-point trajectory (jerky path) and a fit index of 0.5 corresponds to the generation of a maximally smooth point-to-point trajectory. A fit index of 0.5–0.75 was found to correspond to the generation of a non-smooth co-articulated movement (*ABC* or *CDA*), whereas a fit index higher than 0.75 was found to correspond to the generation of a smooth co-articulated movement. A fit index of 1 corresponds to the generation of maximally smooth, curved trajectory between targets *ABC* or *CDA*. Figure 3B depicts representative paths and their respective velocity profiles in different scanning days along with their computed fit indices.

#### COMP condition

As neighboring segments could not be co-planned in the COMP condition (the subjects were instructed to reach a full stop after the completion of each targets' pair), the “global planning” model was applied to each of the four target pairs (*AB*, *BC*, *CD*, and *DA*) assuming a single via-point residing mid-way between each target pair (Flash and Hogan, 1985). In order to attain a fit index of 0.5 when connecting two adjacent targets with a perfect straight point-to-point trajectory (as was attained in the SEQ and REV conditions), the fit index was divided by two (otherwise ending up with a fit index of 1 corresponding to a maximally smooth, curved trajectory).

### Target configuration II

#### SEQ and REV condition

With the assumption that neighboring segments are co-planned, the “global-planning” model was applied to one movement element (*BCD*) assuming a single via-point residing mid-way between the targets as explained for “*Target configuration I—SEQ and REV conditions*.” A fit index was also computed for segment *AB* and segment *DA* as explained in “*Target configuration I—COMP condition*.”

#### COMP condition

As for “*Target configuration I—COMP condition*,” the “global-planning” model was applied to the four target pairs (*AB*, *BC*, *CD*, and *DA*) assuming a single via-point residing mid-way between each target pair and the fit index of each of the four segments was divided by two (otherwise ending up with a fit index of 1 corresponding to a maximally smooth, curved trajectory).

#### Whole-trial smoothness index

As we aimed at testing whether there is a correlation between *whole-trial* brain activation and *whole-trial* motion smoothness, a whole-trial fit index was computed, both for target configuration I and target configuration II. The whole-trial fit index was computed as a simple mean of the fit indices computed for the different segments within a trial.

### Assessing correlation between activation and performance gains

Three types of analyses were performed: condition, pooled and group:

#### Condition (individual) analysis

The motion feature (mean tangential velocity or motion smoothness) was computed for each trial (a sub-block) in each of the three tested conditions and scanning days and the correlation coefficient between the amount of activation in each ROI and the motion feature value was computed [hence, ending up with nine data sets for each of the five subjects, each data set consisting of 16 points (4 blocks * 4 sub-blocks)].

#### Pooled analysis

The nine datasets of each subject were pooled (hence, ending up with one data set for each subject) and the correlation coefficient between the pooled amount of activation and the pooled motion feature values was computed.

#### Group analysis

For each subject the pooled amount of activation was normalized by the highest amount of activation. For trial mean velocity, the pooled trial mean velocity value normalized by the highest trial mean velocity value. The pooled whole-trial smoothness index remained untouched as it is already a normalized index (see *Assessing motion smoothness*). Later, the correlation coefficient between the five subjects' pooled amount of normalized activation and the pooled motion feature was computed.

The rational for conducting three types of analysis was to detect a correlation between brain activation and various low level and high level motion features which may be found in one analysis type but absent in another type due to a significant change in the amount of activation between different conditions and scanning days [e.g., a weak correlation between amount of activation and a low level motion feature found in a condition-based analysis type which is not found in a pooled analysis due to the existence of a strong correlation between amount of activation and a high level motion feature whose level slowly changes throughout training (masking effect)].

## Results

### Co-articulation for adjacent motion elements is accompanied by higher mean velocity and lower motion duration

**Figure 4:**
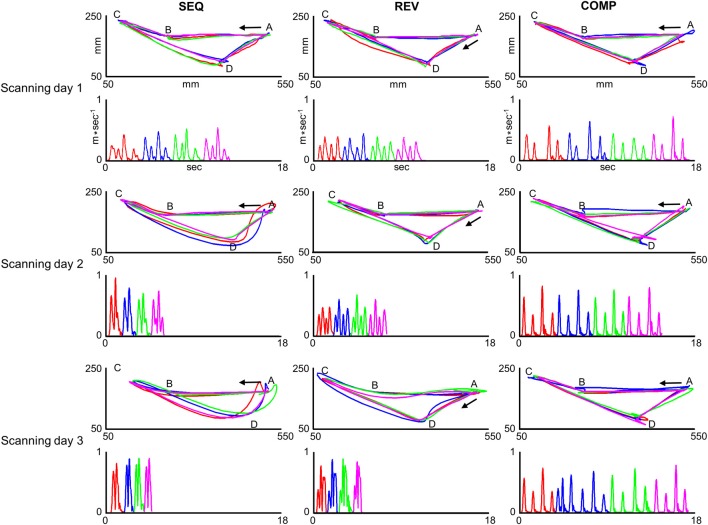
**Trajectories generated inside the MRI scanner by one co-articulating subject (subject #3)**. For each training condition (SEQ, REV, and COMP) and scanning day (scanning day 1, 2, and 3) the red, blue, green, and magenta plots depict four representative paths (upper panels) and their respective tangential velocity profiles (lower panels) generated in two consecutive sub blocks. In order to allow for a comfortable view of the movement velocity profile, the inter sub-block delay was removed and the four movements' velocity profiles are shown in a consecutive manner. The arrow depicts movement direction. The paths generated in the SEQ and REV conditions became smoother with practice whereas the paths generated in the COMP condition remained straight throughout the experiment sessions.

Two subjects (out of three) who practiced target configurations I co-articulated by the end of the last training session in the SEQ and REV conditions. One subject (out of two) who practiced target configurations II co-articulated by the end of the last training session in the SEQ condition and both subjects did not co-articulate in the REV condition by the end of the last training session. Figure 4 depicts paths (upper plots) and their respective velocity profiles (lower plots) generated inside the MRI scanner by a co-articulating subject (subject #3) who practiced target configuration I while performing on the SEQ (leftmost plots), REV (middle plots), and COMP (rightmost plots) conditions before (scanning day 1), during (scanning day 2), and after (scanning day 3) extensive practice.

For the first training session in the SEQ condition, the subject generated four straight point-to-point movements each with a bell-shaped velocity profile (SEQ, Scanning day 1). With continued practice, the paths connecting the first and second pairs of movements (*AB*;*BC* and *CD*;*DA*, respectively) became partially curvilinear with double-peaked velocity profiles (SEQ, Scanning day 2). By the end of the last training session, the prototypical straight paths disappeared and two curved co-articulated paths have emerged, the first with a bell-shaped velocity profile and the second with a double-peaked velocity profile (SEQ, Scanning day 3). For the REV condition, the subject generated four straight point-to-point paths each with a bell-shaped velocity profile both in the first and second training sessions (REV, scanning days 1 and 2, respectively). By the end of the last training session (REV, scanning day 3) the paths connecting target pairs *AB* and *BC* became partially curvilinear with a double-peaked velocity profile and the paths connecting target pairs *CD* and *DA* became curvilinear with a bell-shaped velocity profile. For the COMP condition, the paths connecting all target pairs remained straight throughout practice and were accompanied by a roughly bell-shaped velocity profile.

For the COMP condition, a moderate increase in the trial mean velocity was found for one subject (22%, *p* < 0.05) and no change in trial mean velocity was found for the remaining four subjects throughout the training sessions (*p* > 0.05). For all the five subjects practicing the COMP condition no change in the trial movement duration was found throughout practice (*p* > 0.2) (Table 1).

**Table 1:** **Dependency of trial duration and trial mean velocity on the acquisition of the co-articulation motion strategy**.

**Subject #**	**Condition**	**Movement duration [s]**			**Movement velocity [m/s]**		
		**First scanning day**	**Last scanning day**	**Mean change**	**First scanning day**	**Last scanning day**	**Mean change**
1	SEQ^*^	2.53 ± 0.27	1.33 ± 0.16	−47%^**^	0.11 ± 0.01	0.27 ± 0.04	+145%^**^
		(2.68)	(1.26)		(0.11)	(0.28)	
	REV	2.36 ± 0.15	1.67 ± 0.12	−29%^**^	0.12 ± 0.01	0.21 ± 0.02	+75%^**^
		(2.3)	(1.78)		(0.12)	(0.19)	
	COMP	3.80 ± 0.63	3.64 ± 0.29	−4%	0.08 ± 0.01	0.10 ± 0.01	+25%
		(4.8)	(3.94)		(0.06)	(0.09)	
2	SEQ	3.47 ± 0.56	2.02 ± 0.27	−41%^**^	0.11 ± 0.01	0.17 ± 0.01	+54%^**^
		(3.14)	(1.88)		(0.12)	(0.18)	
	REV	3.16 ± 0.47	2.44 ± 0.36	−22% ^*^	0.12 ± 0.02	0.15 ± 0.02	+25%^**^
		(2.34)	(2.7)		(0.15)	(0.13)	
	COMP	3.68 ± 0.37	3.91 ± 0.15	+6%	0.09 ± 0.01	0.10 ± 0.01	+11%
		(4.1)	(3.78)		(0.09)	(0.08)	
3	SEQ^*^	2.91 ± 0.41	0.98 ± 0.23	−66%^**^	0.12 ± 0.01	0.39 ± 0.06	+225%^**^
		(3.44)	(0.8)		(0.10)	(0.42)	
	REV	2.80 ± 0.26	1.14 ± 0.12	−59%^**^	0.13 ± 0.01	0.34 ± 0.03	+161%^**^
		(2.36)	(1.14)		(0.15)	(0.33)	
	COMP	4.12 ± 0.32	4.06 ± 0.55	−1%	0.09 ± 0.01	0.11 ± 0.01	+22%^**^
		(4.23)	(4.16)		(0.08)	(0.11)	
4	SEQ	1.96 ± 0.21	1.54 ± 0.10	−21%^**^	0.15 ± 0.02	0.18 ± 0.01	+20%^**^
		(2.24)	(1.52)		(0.13)	(0.18)	
	REV	2.05 ± 0.24	1.79 ± 0.21	−23%^*^	0.14 ± 0.02	0.15 ± 0.02	+7%
		(1.92)	(1.56)		(0.14)	(0.19)	
	COMP	3.53 ± 0.30	3.72 ± 0.11	+5%	0.09 ± 0.01	0.08 ± 0.01	−11%^*^
		(3.74)	(3.66)		(0.12)	(0.09)	
5	SEQ	2.65 ± 0.70	2.44 ± 0.29	−8%	0.12 ± 0.02	0.11 ± 0.01	−8%
		(3.42)	(2.08)		(0.09)	(0.13)	
	REV	2.83 ± 0.55	3.16 ± 0.19	+11%	0.11 ± 0.02	0.09 ± 0.01	−18%
		(2.1)	(3.08)		(0.13)	(0.09)	
	COMP	3.60 ± 0.27	3.21 ± 0.68	−10%	0.09 ± 0.01	0.09 ± 0.02	0%
		(3.56)	(3.66)		(0.08)	(0.12)	

The asterisk denotes conditions in which subjects smoothly co-articulated (whole trial smoothness index > 0.75), by the end of the last training session. For each trial in each training condition, the motion duration and mean velocity were computed and a significant change between their values in the first and last training session was tested (

* p < 0.05 two-tailed t-test,

** *p < 0.01 two-tailed t-test). The numbers in parentheses in the first scanning day and last scanning day denote motion index value for the first trial in the first scanning day and last trial in the last scanning day, respectively. The acquisition of the co-articulation motion strategy was accompanied by a significant increase in the trial mean velocity and a significant decrease in the trial movement duration*.

For the SEQ and REV conditions, the mean increase in the trial mean velocity was higher for subjects who made a transition throughout training from the generation of straight trajectories to the generation of curved ones than for subjects who continued generating straight point-to-point trajectories throughout training (130 and 23% for the co-articulating and non co-articulating subjects, respectively, in the SEQ condition and 118 and 5% for the co-articulating and non co-articulating subjects, respectively, in the REV condition). The increase in trial mean velocity found for the co-articulating subjects was accompanied by a significant reduction in trial movement duration (44.6 and 24.5% for the co-articulating and non co-articulating subjects, respectively, in the SEQ condition and 44.0 and 18.6% for the co-articulating and non co-articulating subjects, respectively, in the REV condition).

### The amount of activation is not dependent on the amount of practice

**Figure 5:**
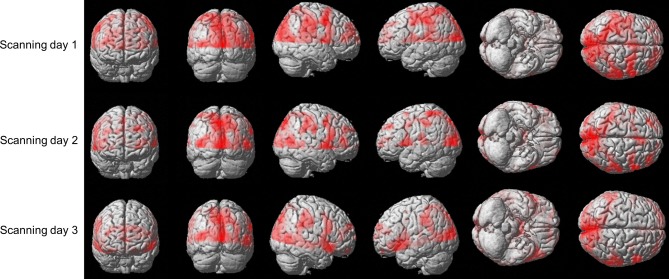
**Brain activation of subject #3 while practicing the SEQ condition in the 3 scanning days**. Depicted is the activation (*t*-stats for the contrast movement vs. rest) in the 12 axial slice (no gap) that were oriented parallel to the bi-commissural plane with the uppermost slice aligned 5 mm below the vertex.

We sought to examine whether the amount of activation in different brain areas, in general, and in the predefined ROIs, in particular, is correlated with the amount of practice. Figure 5 shows the brain activation of one co-articulating subject while practicing the SEQ condition in the 3 scanning days. As is readily seen, performing the task involved activation of the bilateral Calcarine, bilateral Precentral gyrus, bilateral Cuneus, left and right mid temporal lobe, left and right mid occipital lobe, left Postcentral gyrus, right Rolandic operculum, right Precuneus, and right SupraMarginal gyrus.

**Figure 6:**
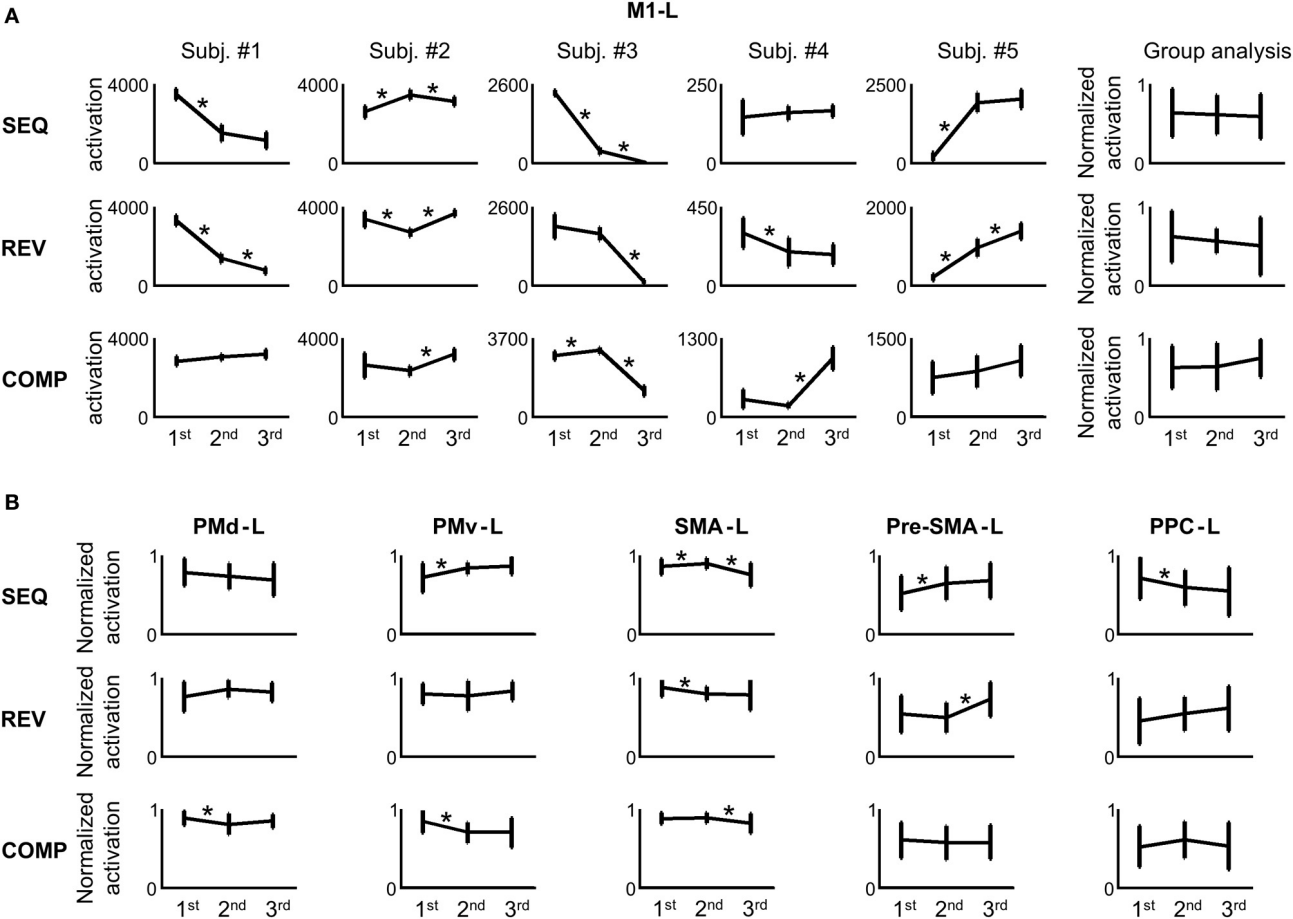
**No correlation between the amount of activation in the different regions of interest (ROIs) and the amount of practice. (A)** For each repetition, the M1-L activation was estimated. The error bars represent error across repetitions. The rightmost panel in each training condition presents the group analysis (the normalized activations of all the subjects in each scanning day against the amount of training). The asterisk denotes a significant change in activation between 2 consecutive scanning days (*p* < 0.05). **(B)** Group analysis (normalized activations of all the subjects in each scanning day against the amount of training) for the PMd-L, PMv-L, SMA-L, pre-SMA-L, and PPC-L.

In order to attain a quantitative measure of the activation, we computed the number of voxels activated in the M1-L arm area (including the wrist, elbow, hand, and fingers, see Materials and Methods) in each condition. Figure 6A depicts the amount of activation in the M1-L arm area of each of the subjects while performing on the three tested conditions in each of the three scanning sessions. As is readily seen, no consistent pattern of dependency of the amount of M1-L activation on the amount of practice was found while performing on the heavily practiced SEQ condition [e.g., for subjects #1 and #3 there was a significant decrease in the amount of activation from scanning day 1 to scanning day 2 (*p* < 0.01, two tailed *t*-test) whereas for two subjects (#2 and #5) the amount of activation increased significantly from scanning day 1 to scanning day 2 (*p* < 0.01, two tailed *t*-test) and for one subject (subject #4) no significant change in the activation was found (*p* > 0.05, two-tailed *t*-test)]. No consistent pattern of dependency of the amount of M1-L activation on the amount of practice was also found while performing on the REV and COMP transfer conditions (Figure 6A, mid and lower plots). The lack of activity (i.e., zero voxels activated) found in some brain areas (e.g., subject # 3, SEQ condition, scanning day 3) results from the high threshold of statistical significance imposed on the data and does not imply that these brain areas are not involved in planning/executing the task after it has been over learned.

In order to perform a group analysis we normalized, for each subject and tested condition, the amount of M1-L activation recorded in the 3 scanning days by the highest amount of activation. Thus, five normalized data sets were produced—one for each subject. Each data set was composed of three subsets—one for each condition (e.g., for subject #1 in the SEQ condition, we normalized the amount of activation computed in the 3 scanning days by the highest amount of activation, recorded on scanning day 1). Next, we pooled the normalized activations of the five subjects in each of the three tested conditions and looked for a dependency of the M1-L amount of activation on the amount of practice. Overall, no significant change in M1-L pooled activation was found between scanning day 1 and scanning day 2 and between scanning day 2 and scanning day 3 for each of the three tested conditions (*p* > 0.05) (Figure 6A, upper, mid, and lower rightmost plots). Given that the subjects have gone through similar amount of practice, these results imply that the activity in the contralateral M1 arm area was not dependent on the amount of practice *per se*.

No consistent pattern of change in the amount of activation throughout the 3 scanning days among conditions was found for any of the other tested motor areas: the contralateral PM (PMd-L and PMv-L), contralateral SMA (SMA-L proper and pre-SMA-L), and the contralateral PPC (PPC-L) (Figure 6B). For individual plots of amount of activation vs. amount of practice see Figure S1 (supplementary material). The same results were found for the analog ipsilateral regions.

### The correlation between the amount of individual M1-L activation and trial mean velocity is modulated by a performance-related effect

**Figure 7:**
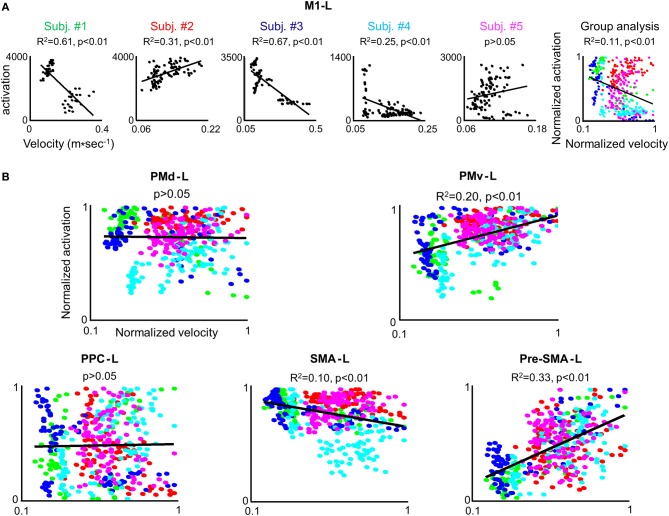
**The correlation between the individual amount of M1-L activation and trial mean velocity is modulated by a performance-related effect. (A)** For each subject the amount of M1-L activation vs. trial mean velocity while training on the three tested conditions in the 3 scanning days is depicted. The correlation coefficient and the significance level (R^2^ and *p*-value, respectively)between the two descriptors are indicated and the regression line is depicted. The rightmost plot depicts group analysis. The green, red, blue, cyan, and magenta colored dots denote normalized data from subject #1, #2, #3, #4, and #5, respectively. **(B)** Group analysis for PMd-L, PMv-L, SMA-L, pre-SMA-L, and PPC-L. Color coding as for **(A)**.

In order to test whether the amount of activation in the different ROIs is correlated with the trial mean velocity we conducted the condition-based analysis (see Materials and Methods). For 22 conditions (out of 45) a correlation was found between M1-L activation and trial mean velocity (*p* < 0.05). As expected, in all of these conditions, the correlation was found to be both strong and positive (Table 2). Next, we aimed at testing the existence of a correlation between amount of activation and trial mean velocity, *irrespective of the training condition and amount of practice*. To that end, we conducted the pooled-based analysis—testing whether the pooled amount of M1-L activation of each subject correlates with its corresponding pooled trial mean velocity. Surprisingly, no consistent pattern of correlation between pooled amount of M1-L activation and trial mean velocity was found among subjects (Figure 7A, five leftmost plots, black solid line); the correlation between the two descriptors was found to be strong and negative for two subjects [*R*^2^ = 0.61 and *R*^2^ = 0.67 for subject #1 and #3, respectively), weak and *negative* for one subject (*R*^2^ = 0.25 for subjects #4), weak and *positive* for one subject (*R*^2^ = 0.31 for subject #2) and non-significant for one subject (*p* > 0.05 for subject #5)].

**Table 2:** **Coefficients of determination for the correlation between individual amount of M1-L activation and trial mean velocity**.

**Subject #**	**Day**	**SEQ**	**REV**	**COMP**
1	1	0.71↑	^___^	0.67↑
	2	^___^	0.93↑	^___^
	3	0.79↑	^___^	0.45↑
2	1	0.60↑	^___^	^___^
	2	^___^	0.88↑	0.59↑
	3	0.58↑	0.86↑	^___^
3	1	0.60↑	0.70↑	^___^
	2	^___^	^___^	0.49↑
	3	^___^	0.82↑	0.53↑
4	1	^___^	0.60↑	^___^
	2	0.44↑	0.50↑	^___^
	3	^___^	^___^	^___^
5	1	0.54↑	^___^	^___^
	2	^___^	0.23↑	^___^
	3	0.42↑	^___^	0.77↑

*For each tested condition and scanning day a correlation coefficient between the individual amount of M1-L activation and trial mean velocity was computed. The values denote R-square statistic (multiple linear regression using least squares, p < 0.05). ↑ A positive correlation. Underscore, A condition in which no correlation between the two descriptors was found (p > 0.05). For 22 conditions (out of 45) a correlation between the two descriptors was found (p < 0.05)*.

Overall, the non-consistent pattern of correlation between pooled M1-L activation and trial mean velocity among subjects resulted in a very weak correlation between the two descriptors when conducting the group analysis (*R*^2^ = 0.11) (Figure 7A, rightmost plot).

We performed the three types of analysis on the PMd-L data. The condition-based analysis showed that velocity coding in PMd-L is less prominent than in M1-L; a correlation between the two descriptors was found in only 10 (out of 45) conditions. As found for M1-L, in all but one of the cases the correlation was found to be both strong and positive (Supplementary Table 1). No correlation between the two descriptors was found while performing the pooled analysis or group analysis (Figure 7B, left upper plot).

No correlation between the two descriptors was found for the PMv-L, SMA-L, pre-SMA-L, and PPC-L in any of the conditions when conducting the condition-based analysis (*p* > 0.05) (Figure 7B, right upper plot and three lower plots). A weak correlation was found between the descriptors in the PMv (*R*^2^ = 0.2), contralateral SMA (*R*^2^ = 0.1) and contralateral pre-SMA (*R*^2^ = 0.33) when performing group analysis. For individual plots of activation level vs. trial mean velocity see Figure S2 (supplementary material).

Next, we sought to examine whether the decrease in correlation power (in M1-L), or its disappearance (in PMd-L) when moving from the condition-based analysis to the pooled analysis, results from a significant change in the baseline activation in the different scanning days and whether this change in amount of activation correlates with a high level performance gains—shifting from the generation of straight point-to-point trajectories to the generation of curved, smooth trajectories throughout training. To that end, a whole-trial fit index was computed for each subject in each tested condition and scanning day (see Materials and Methods).

### Smoothness evolution throughout practice

#### Coarticulating subjects (#1, #3, and #4)

For subject #3 the whole-trial fit index in the SEQ condition increased significantly from scanning day 1 (0.42 ± 0.07) to scanning day 2 (0.67 ± 0.11) (*p* < 0.01) with no significant change in scanning day 3 (0.80 ± 0.05), which corresponds to a rapid transition from the generation of four straight point-to-point trajectories in scanning day 1 to the generation of two curved, smooth trajectories in scanning days 2 and 3 (Supplementary Table 2). The same pattern was found for subject #1 (0.51 ± 0.03, 0.76 ± 0.07, 0.78 ± 0.05 for scanning day 1, 2, and 3, respectively) and subject # 4 (0.39 ± 0.07, 0.56 ± 0.05, 0.59 ± 0.06 for scanning day 1, 2, and 3, respectively).

For all the three subjects, the whole-trial fit index in the REV condition increased significantly from scanning day 1 to scanning day 2 onward to scanning day 3 but has not reached the high performance gains attained in the SEQ condition by the end of the last training session implying that performance gains in the transfer (REV) condition lagged performance gains in the trained (SEQ) condition.

No significant change in the whole-trial fit index was found throughout training while practicing the COMP condition (*p* > 0.05).

#### Non coarticulating subjects (#2 and #5)

For subject #2, the fit index in the SEQ condition has not changed significantly from scanning day 1 (0.40 ± 0.05) to scanning day 2 (0.43 ± 0.05) but increased significantly in scanning day 3 (0.51 ± 0.06) implying a shift from the generation of non-straight (jerky) four point-to-point trajectories to the generation of four straight point-to-point trajectories. No significant change in the whole-trial fit index was found in the REV condition (0.4 ± 0.09, 0.4 ± 0.05, and 0.39 ± 0.06) and COMP condition (0.33 ± 0.03, 0.33 ± 0.02, and 0.32 ± 0.03) implying that the trajectories remained non-straight (jerky) throughout training. The same qualitative results were found for subject #5 apart from the finding that his point-to-point movements in the SEQ condition remained non straight (jerky) throughout training.

#### Individual amount of M1-L activation is not correlated with motion smoothness

**Figure 8:**
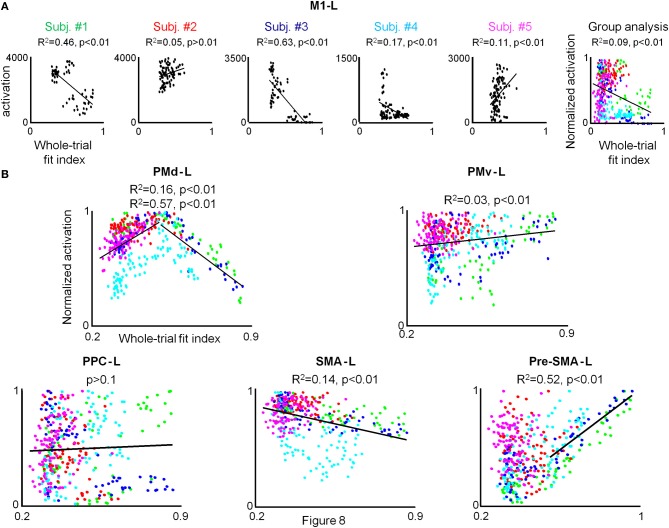
**A strong correlation between the individual amount of activation and whole-trial fit index is found only for PMd-L and preSMA-L. (A)** For each subject the pooled amount of M1-L activation vs. motion smoothness whole-trial fit index while training on the three tested conditions in the 3 scanning days is depicted. The correlation coefficient and the significance level (*R*^2^ and *p*-value, respectively) between the two descriptors are indicated and the regression line is depicted. The rightmost plot depicts group analysis. The green, red, blue, cyan, and magenta colored dots denote normalized data from subject #1, #2, #3, #4, and #5, respectively. **(B)** Group analysis for PMd-L, PMv-L, SMA-L, pre-SMA-L, and PPC-L. Color coding as for **(A)**.

No correlation between the amount of M1-L activation and motion smoothness was found for each of the five participants when conducting the condition-based analysis. In order to test whether the lack of observed correlation between the two descriptors results from the weak dependence of the amount of M1-L activation on motion smoothness in this area (thus, precluding the reflection of the small motion smoothness variations in a given condition in the amount of activation), we conducted the pooled analysis. For the three co-articulating subjects (#1, #3, and #4), the increase of the fit index from 0.3 (generation of jerky point-to-point trajectories) to 0.85 (generation of a smooth curved trajectory) was found to be *negatively* correlated with the amount of activation in the M1-L arm area (*R*^2^ = 0.45, *R*^2^ = 0.63, and *R*^2^ = 0.17 for subject #1, #3, and #4, respectively) (Figure 8A, Subj. #1, #3, and #4). For both of the non-co-articulating subjects (#2 and #5) for whom no substantial change in the motion smoothness was found throughout training, no consistent correlation between the amount of M1-L activation and motion smoothness was found (Figure 8A, Subj. #2 and #5); for subject #2 the increase of the fit index from 0.28 to 0.62 was not significantly correlated with the amount of M1-L activation (*p* > 0.1) while for subject #5 the increase of the fit index from 0.25 to 0.58 was very weakly *positively* correlated with the amount of M1-L activation (*R*^2^ = 0.11).

The weak dependence of M1-L activation on motion smoothness, which was expressed in the existence of a correlation between the two descriptors only when substantial changes in motion smoothness were found (i.e., between different sessions) was also manifested while conducting the group analysis (*R*^2^ = 0.09, *p* < 0.01).

#### The individual amount of PMd-L activation is highly correlated with motion smoothness

Conducting the group analysis has revealed no correlation between the amount of group PMd-L activation and motion smoothness (*p* > 0.05). However, in depth examination of the scatter plot revealed a consistent pattern of correlation between the amount of PMd-L activation and motion smoothness across subjects; for all conditions in which the subjects shifted from generating jerky trajectories to generating straight point-to-point trajectories the correlation between the amount of PMd-L activation and motion smoothness was non-significant or positive whereas for all conditions in which subjects shifted from generating straight point-to-point trajectories to generating smooth, curved trajectories the correlation between the two descriptors was found to be non-significant or negative (Supplementary Table 3).

Conducting the pooled subject data analysis has resulted in similar qualitative findings for each of the five subjects. An increase of the fit index from 0.2 (generation of four jerky point-to-point trajectories) to 0.5 (generation of four straight point-to-point trajectories) was *positively* correlated with the amount of PMd-L activation (*R*^2^ = 0.4, *R*^2^ = 0.06, *R*^2^ = 0.78, *R*^2^ = 0.66, and *R*^2^ = 0.44 for subject #1, #2, #3, #4, and #5, respectively) whereas an increase of the fit index from 0.5 to 0.9 (generation of two highly smooth, curved trajectories) was *negatively* correlated with the amount of PMd-L arm area activation (*R*^2^ = 0.87, *R*^2^ = 0.67, *R*^2^ = 0.88, and *R*^2^ = 0.53 for subject #1, #2, #3, and #4, respectively) (see Figure S3 in the supplementary material).

Conducting the group analysis resulted in similar qualitative results (Figure 8, PMd-L). A positive correlation between the pooled PMd-L activation and motion smoothness was found when subjects shifted from generating jerky point-to-point trajectories to generating straight trajectories (*R*^2^ = 0.16) while a negative correlation between the two descriptors was found when subjects shifted from generating straight point-to-point trajectories to the generation of curved, smooth trajectories (*R*^2^ = 0.57).

#### The individual amount of preSMA-L activation increases with movement co-articulation

Conducting the group analysis has revealed no correlation between the amount of group preSMA-L activation and motion smoothness (*p* > 0.05). However, thorough inspection of the scatter plot has revealed that whereas for all conditions in which subjects shifted from generating jerky point-to-point trajectories to generating straight point-to-point trajectories no correlation between individual amount of pre-SMA-L activation and motion smoothness was found, a strong positive correlation between the two descriptors is found for all conditions in which subjects shifted from generating straight point-to-point trajectories to generating smooth, curved trajectories (Supplementary Table 4). Conducting the pooled analysis has resulted in similar qualitative results for each of the five subjects; an increase of the fit index from 0.3 (generation of four jerky point-to-point trajectories) to 0.5 (generation of four straight point-to-point trajectories) was not found to correlate with the individual amount of pre-SMA-L activation whereas an increase of the fit index from 0.5 to 0.9 (generation of two highly smooth curved trajectories) was found to be *positively* correlated (see Figure S3 in the supplementary material). As expected, the group analysis has revealed a positive correlation between the amount of pre-SMA-L activation and motion smoothness when subjects shifted from generating straight point-to-point trajectories to generating curved, smooth trajectories (*R*^2^ = 0.52) (Figure 8B, pre-SMA-L).

No correlation between the two descriptors was found for the PMv-L, SMA-L, and PPC-L while conducting the condition-based, pooled, and group analysis.

## Discussion

In this study we aimed at unraveling the nature of the change in activation that takes place in different motor cortical areas while training on a new motor task, its relation to different kinematic attributes of the movement and the role it plays in the acquisition, representation and implementation of a newly acquired motor skill (results from sub-cortical areas and the Cerebellum will be presented in a different paper). Specifically, we sought to unravel whether the engagement of the different motor cortical areas during learning of a new motor task correlates or depends on the existence of learning stages, defined by kinematic performance indices. In order to relate to previous studies which showed the influence of practice on activation in different motor cortical areas, we aimed at attaining a qualitative measure of the activation in these regions. As only five subjects took place in this study and the experimental paradigm made use of two target configurations, we consider this work as a multi case study and implemented only first level fMRI analysis. However, several activation features were common to all subjects. Firstly, we found that there was no consistent pattern among subjects of dependency of amount of activation on amount of practice (Figure 6). Given that different subjects attained different performance gains by the end of the last training session, this finding is not surprising. Numerous studies have shown that cortical and sub-cortical motor neurons code for various kinematic and dynamic features (Georgopoulos et al., 1982; Kettner et al., 1988; Schwartz et al., 1988; Kalaska and Crammond, 1992; Schwartz and Moran, 1999; Turner et al., 2003) and thus, the influence of the prolonged practice, *per se*, could be modulated by the different levels of performance gains which varied among subjects at any given time point throughout the practice period.

The strong and moderate correlation between the individual amount of activation and trial mean velocity found in M1-L and PMd-L, respectively, were weakened or gone when conducting the pooled and group analysis, respectively (Figure 7). Since many kinematic features of the movement have changed throughout the prolonged training period, changes in amount of activity due to swings in trial mean velocities may have been be modulated, or even masked by high-level motion features dependent activity, such as trajectory shape and motion smoothness. Thus, the subtle changes in activation, correlated with the trial mean velocity, may have been superimposed on a baseline activity which is largely dependent on high level performance gains and may change from session to session. This notion is supported by the finding that a positive correlation between amount of activation and trial mean velocity was found in M1-L, and to a lesser extent in PMd-L when performing the condition-based analysis for which performance-related activation changes have smaller effects on the overall amount of activation (due to the small performance gain changes in a given condition and session). The finding that the strongest correlation between amount of activation and mean velocity was found for M1-L (Table 2), and to a lesser extent for PMd-L (Supplementary Table 1) supports earlier findings that M1-L region encodes low level motion kinematic features (Moran and Schwartz, 1999a; Ifft et al., 2011).

The amount of activation in the PMd-L was found to correlate with trajectory shape; the amount of activation in this region increased significantly when subjects shifted from generating jerky point-to-point trajectories to generating straight point-to-point trajectories and later decreased significantly when subjects shifted from generating straight point-to-point trajectories to the co-articulation of adjacent motion segments into a smooth, curved trajectory (Figure 8). These findings were consistent for both the condition-based, pooled and group analysis. Overall, the weak correlation found between the individual amount of PMd-L activation and mean velocity and the strong correlation found between the individual activation in this area and the global shape of the trajectory (jerky, straight, curved) and its smoothness implies its role in coding higher level attributes of the motion. It is not likely that the maximal activity found while subjects generated four straight point-to-point trajectories (both in the SEQ, REV, or COMP conditions) was caused by higher levels of muscle activity as the neural activity in trials in which subjects generated jerky movements was smaller, although it was expected to be higher (due to the generation of small corrections movements accompanied by acceleration and deceleration phases). However, in order to ascertain that the maximal activity registered while subjects generated straight point-to-point trajectories is not a byproduct of low level, dynamic features of the movement, it would be of interest to record the EMG activity while conducting a similar experiment.

It was shown lately that the pre-SMA participates in chunking basic motion elements into motor sequences (Nakamura et al., 1998; Kennerley et al., 2004) and is involved in their representation in a non-effector specific manner (Grafton et al., 1998, 2002; Hikosaka et al., 1999, 2002; Turner et al., 2003). All the aforementioned findings related to “discrete” actions, e.g., reach a ball, touch a key, play certain piano notes, etc., and no reference was given to the spatiotemporal characteristics of the action. In our study, the pre-SMA–L activity did not change significantly when subjects shifted from generating jerky point-to-point trajectories to generating straight point-to-point trajectories, however, a significant increase in activation was found when subjects shifted from generating straight point-to-point trajectories to the generation of a smooth, curved trajectory (Figure 8). These findings support the notion that the pre-SMA is involved in the concatenation of basic motion elements and may suggest that it is involved in the generation and representation of novel motor sequences which are composed of motion elements that undergo spatiotemporal changes (by the process of co-articulation) rather than just being sequenced in a different order in each sequence.

In our work, no change in activation was found in SMA-L while shifting from generating straight point-to-point trajectories to generating concatenated, and later curved, smooth trajectories. It was previously shown that this region is involved in coordinating temporal sequences of actions and the formation of sequential procedural memory (Mushiake et al., 1990; Shima and Tanji, 1998; Hikosaka et al., 1999; Sakai et al., 1999; Lee and Quessy, 2003; Bischoff-Grethe et al., 2004) As motion parameters indices (velocity, motion smoothness) computed for the entire training period in our study have indicated that performance did not asymptote by the end of the last training session, it is possible that given additional practice, the memory for motor sequence would be consolidated and a significant change in SMA-L activity throughout practice will be found.

## Limitations of the work and possible extensions

Only five subjects have taken part in the study, thus, the presented findings should be regarded as a proof of concept of the effect of various performance features on the amount of activation in different motor areas, in general, and the modulation of individual M1-L activation by high level motion features, such as smoothness and co-articulation, in particular. Nevertheless, given that our analysis aimed at testing whether the amount of activation in different motor ROIs is correlated with specific, individual performance gains and not on task constraints (such as the amount of practice), which were common to all the subjects, we were able to look for patterns of activity that were common among subjects at different learning stages. For example, although some subjects have generated movements whose corresponding smoothness fit indices spanned only a small portion of the fit index range (subject #2, #5) whereas others have generated movements whose corresponding smoothness fit indices spanned most of the possible range (subject #1, #3, and #4), in all cases the correlation pattern between the tested motion parameters (velocity, smoothness) and the amount of activation was similar.

As to maintain as natural training conditions as possible, no instructions were given to the subjects to refrain from eye movements during hand movement. Given that the motion planning strategy has changed for some subjects throughout training (e.g., shifting from generating jerky point-to-point trajectories to generating straight point-to-point trajectories or shifting from generating straight point-to-point trajectories to generating curved, smooth trajectories), it might be that the eye movement's pattern changed concurrently. Although our ROI in M1 was restricted to the arm area and no correlation is expected to be found between the amount of activation in this region and the number of generated saccades, the eye movements might have had a modulating effect on the amount of activation in this ROI and other tested motor regions.

Finally, no methodology was used to dissociate activation changes due to changes in performance from activation changes due to differences in the actual representation of the task following training. Although the evolving representation of a motor skill should readily manifest itself in overt motion features in abled subjects further experiments are needed to dissociate between the two and there is a need for natural tasks for studying the real correlates of the learning of complex movements.

## Conclusions

Overall, our findings suggest that the amount of activation in M1-L, PMd-L, and pre-SMA-L is modulated by low level and high level performance gains and that individual performance gains and learning stages, as defined by kinematic or dynamic motion features, should be considered whenever relating changes in amount and extent of brain activation to a specific motor task.

### Conflict of interest statement

The authors declare that the research was conducted in the absence of any commercial or financial relationships that could be construed as a potential conflict of interest.

## Supplementary material

Figure S1 **Individual plots of amount of activation vs. amount of practice**. For each subject the PM-L (PMd-L and PMv-L), SMA-L (SMA proper and pre-SMA), and the PPC-L (PPC-L) activation while training on the three tested conditions (SEQ, REV, and COMP) in the 3 scanning days are depicted. The asterisk denotes a significant change in activation between 2 consecutive scanning days (*p* < 0.05).

Figure S2 **Individual plots of amount of activation vs. trial mean velocity**. For each subject the PM-L (PMd-L and PMv-L), SMA-L (SMA proper and pre-SMA), and the PPC-L (PPC-L) amount of activation vs. trial mean velocity while training on the three tested conditions (SEQ, REV, and COMP) in the 3 scanning days is depicted. The red, blue and green symbols denote data from the first, second and third scanning day, respectively. The square, triangle and circle symbols denote data from the COMP, REV, and SEQ condition, respectively. For each subject the correlation coefficient and the significance level (*R*^2^ and *p*, respectively)between amount of activation and trial mean velocity are indicated and the regression line is depicted.

Figure S3 **Individual plots of amount of activation vs. motion smoothness**. For each subject the PM-L (PMd-L and PMv-L), SMA-L (SMA proper and pre-SMA), and the PPC-L (PPC-L) amount of activation vs. motion smoothness whole-trial fit index while training on the three tested conditions (SEQ, REV, and COMP) in the 3 scanning days is depicted. The red, blue, and green symbols denote data from the first, second and third scanning day, respectively. The square, triangle and circle symbols denote data from the COMP, REV, and SEQ condition, respectively. For each subject the correlation coefficient and the significance level (*R*^2^ and *p*, respectively)between amount of activation and motion smoothness whole-trial fit index are indicated and the regression line is depicted.
