# Supplementary figures and images for: The activity in the contralateral primary motor cortex, dorsal premotor and supplementary motor area is modulated by performance gains
Source: Front Hum Neurosci. 2014 Apr 16;8:201. doi: 10.3389/fnhum.2014.00201 (PMC3997032; doi:10.3389/fnhum.2014.00201)

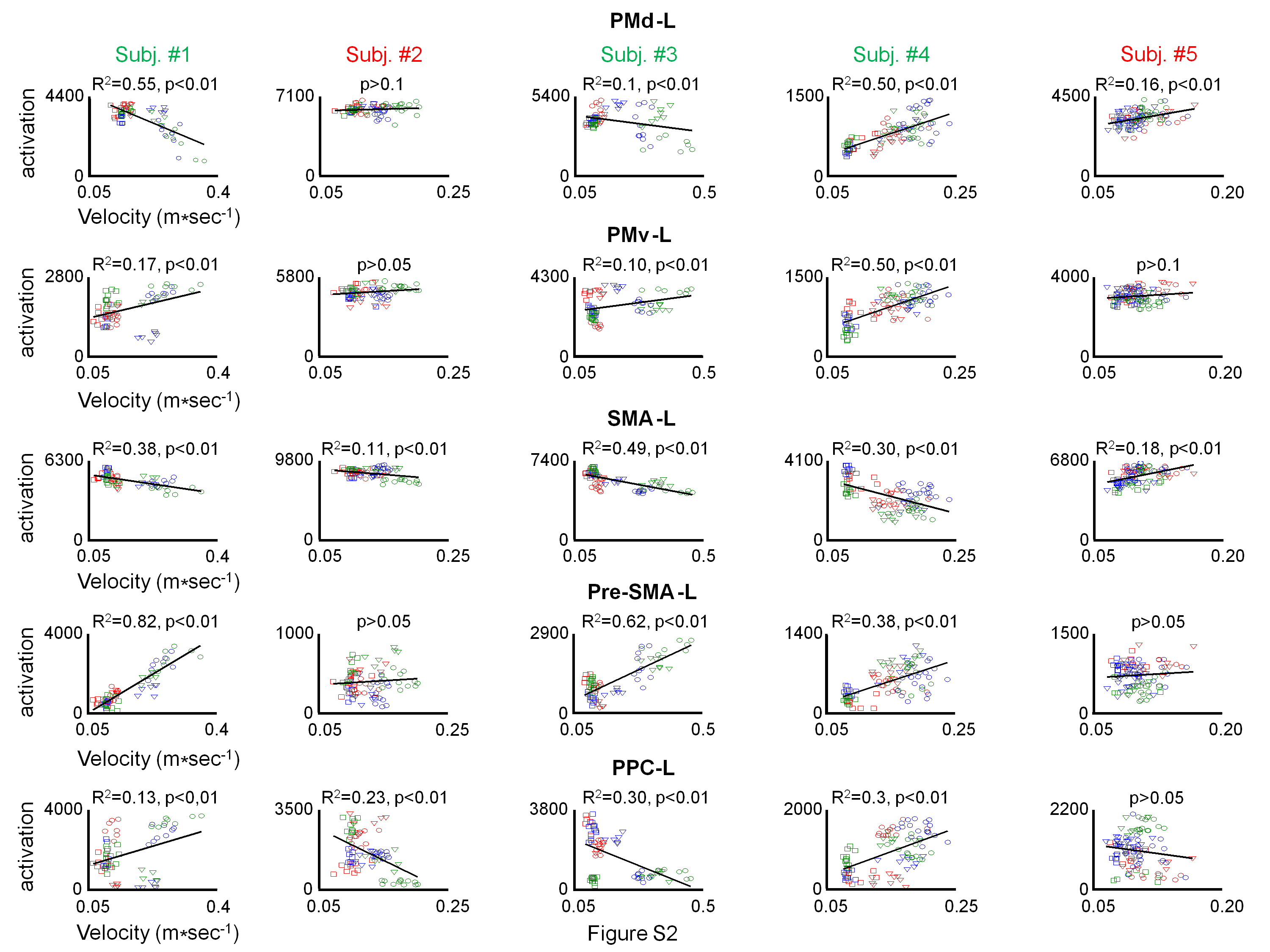

Supplement: Figure S1 — Individual plots of amount of activation vs. amount of practice. For each subject the PM-L (PMd-L and PMv-L), SMA-L (SMA proper and pre-SMA), and the PPC-L (PPC-L) activation while training on the three tested conditions (SEQ, REV, and COMP) in the 3 scanning days are depicted. The asterisk denotes a significant change in activation between 2 consecutive scanning days (p < 0.05). [file Presentation1.ZIP › 80533__Supplementary Figure_2.TIF]

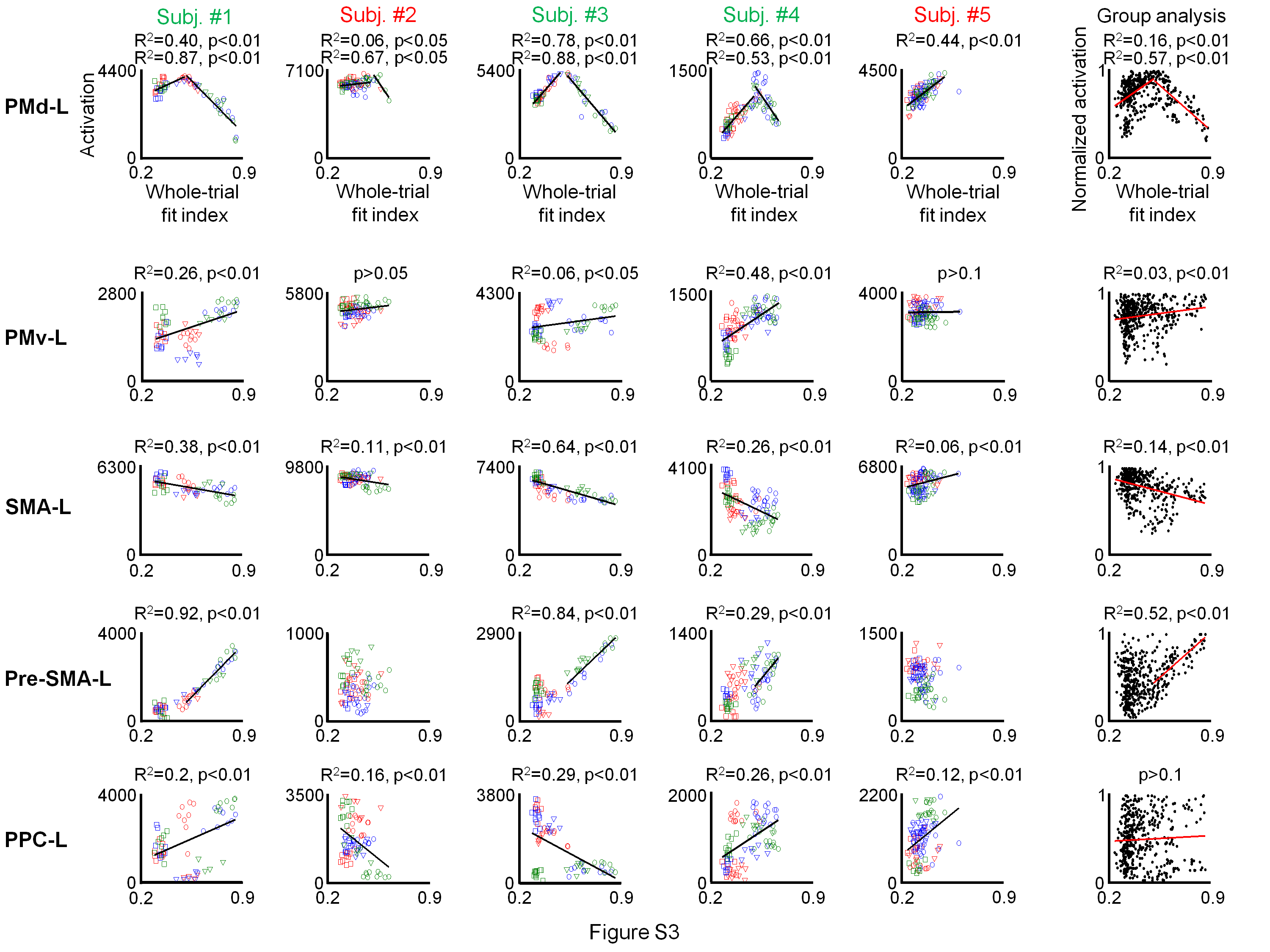

Supplement: Figure S1 — Individual plots of amount of activation vs. amount of practice. For each subject the PM-L (PMd-L and PMv-L), SMA-L (SMA proper and pre-SMA), and the PPC-L (PPC-L) activation while training on the three tested conditions (SEQ, REV, and COMP) in the 3 scanning days are depicted. The asterisk denotes a significant change in activation between 2 consecutive scanning days (p < 0.05). [file Presentation1.ZIP › 80533__Supplementary Figure_3.TIF]

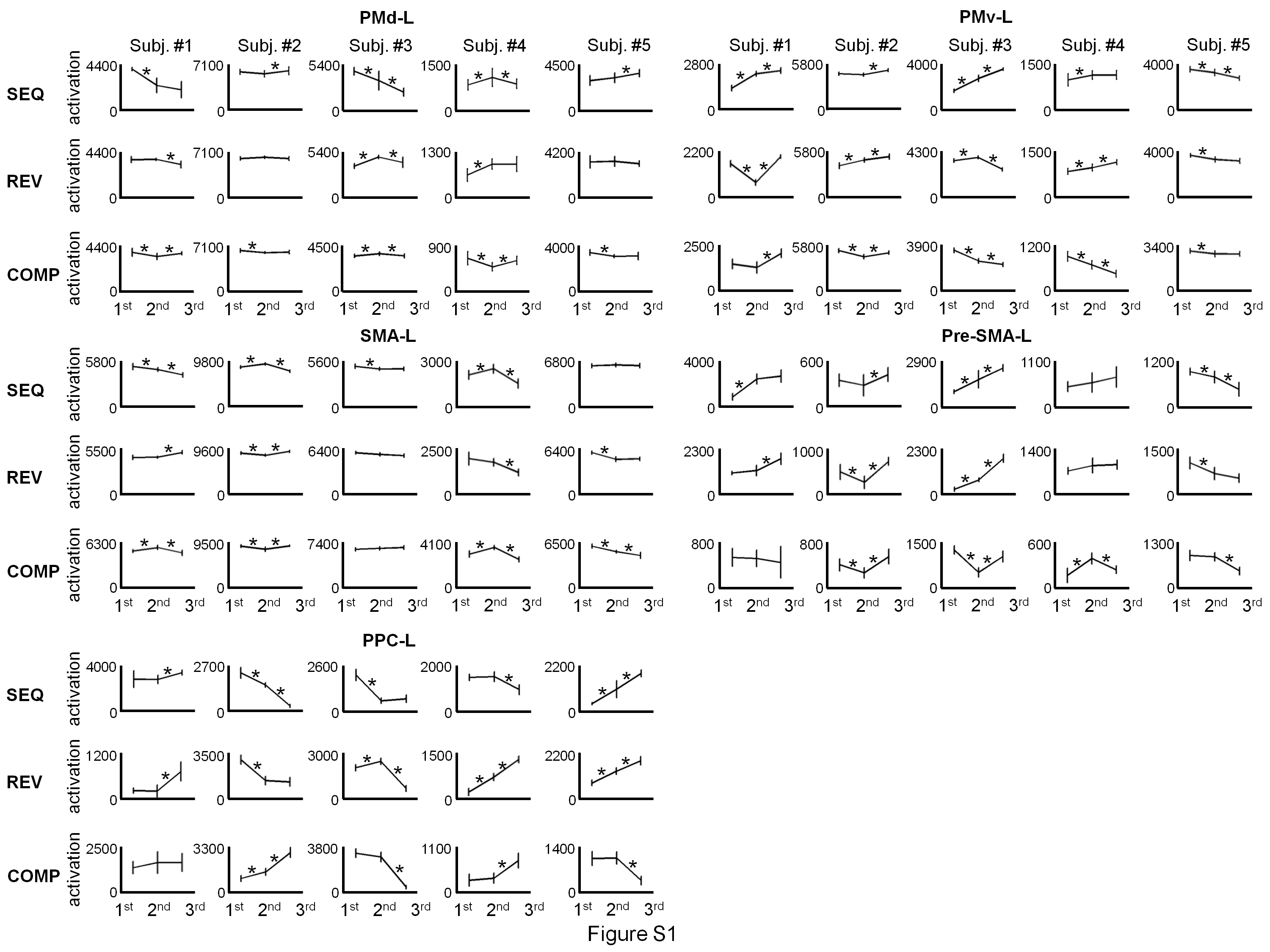

Supplement: Figure S1 — Individual plots of amount of activation vs. amount of practice. For each subject the PM-L (PMd-L and PMv-L), SMA-L (SMA proper and pre-SMA), and the PPC-L (PPC-L) activation while training on the three tested conditions (SEQ, REV, and COMP) in the 3 scanning days are depicted. The asterisk denotes a significant change in activation between 2 consecutive scanning days (p < 0.05). [file Presentation1.ZIP › 80533__Supplementary Figure_1.TIF]
