# Supplementary material for: The activity in the contralateral primary motor cortex, dorsal premotor and supplementary motor area is modulated by performance gains
Source: Front Hum Neurosci. 2014 Apr 16;8:201. doi: 10.3389/fnhum.2014.00201 (PMC3997032; doi:10.3389/fnhum.2014.00201)
Supplement: Supplementary file 2 [file DataSheet1.PDF]

Subject #	Day	SEQ	REV	COMP
1	1	—	—	—
	2	—	—	—
	3	0.88↓	—	—
2	1	—	—	0.53↑
	2	—	0.79↑	—
	3	—	—	0.79↑
3	1	—	0.83↑	—
	2	—	—	—
	3	—	—	0.33↑
4	1	—	0.87↑	—
	2	—	0.89↑	—
	3	—	—	—
5	1	—	—	0.75↑
	2	—	—	0.44↑
	3	—	—	—

Supplementary Table 1: A positive correlation between individual amount of PMd-L activity and trial mean velocity. For each tested condition and scanning day a correlation coefficient between individual amount of activation and trial mean velocity was computed. The values denote R-square statistic (multiple linear regression using least squares, $p<0.05$). ↑ A positive correlation**.** ↓ A negative correlation**. Underscore** A condition in which no correlation was found ($p>0.05$). For 10 conditions (out of 45) a high correlation was found between the two descriptors ($p<0.05$). The correlation between the two descriptors was found to be positive for nine of the ten conditions.

subject #	Condition	Smoothness fit index<br>First scanning day	 Last scanning day	 mean change
1	SEQ	0.51 ± 0.03 (0.51)	0.78 ± 0.05 (0.82)	+52% **
	REV	0.54 ± 0.04 (0.58)	0.67 ± 0.07 (0.67)	+24% *
	COMP	0.32 ± 0.02 (0.35)	0.33± 0.02 (0.33)	+3%
2	SEQ	0.40 ± 0.05 (0.35)	0.51 ± 0.06 (0.62)	+27% **
	REV	0.40 ± 0.09 (0.37)	0.39 ± 0.06 (0.39)	-2%
	COMP	0.33 ± 0.03 (0.33)	0.32 ± 0.03 (0.35)	-3%
3	SEQ	0.42 ± 0.07 (0.43)	0.80 ± 0.05 (0.82)	+90% **
	REV	0.34 ± 0.02 (0.39)	0.60 ± 0.04 (0.61)	+76% **
	COMP	0.32 ± 0.01 (0.31)	0.31 ±0.01 (0.30)	-3%
4	SEQ	0.39 ± 0.07 (0.32)	0.59 ± 0.06 (0.65)	+51% **
	REV	0.37 ± 0.04 (0.38)	0.54 ± 0.06 (0.57)	+45% **
	COMP	0.32 ± 0.03 (0.32)	0.31 ± 0.01 (0.36)	-3%
5	SEQ	0.31 ± 0.04 (0.25)	0.39 ± 0.03 (0.41)	+25% **
	REV	0.33 ± 0.08 (0.42)	0.34 ± 0.04 (0.31)	+3%
	COMP	0.29 ± 0.03 (0.25)	0.30 ± 0.03 (0.30)	+3%

Supplementary Table 2: Evolution of motion smoothness in different scanning days and tested conditions. For each trial in each training condition, the smoothness fit index was computed and a significant change between its values in the first and last training session was tested (* $p<0.05$ two-tailed t-test, ** $p<0.01$ two-tailed t-test). The numbers in parentheses in the first scanning day and last scanning day denote smoothness fit index for the first trial in the first scanning day and last trial in the last scanning day, respectively.

Subject #	Day	SEQ		REV		COMP	
		**0.2 → 0.5**	**0.5 → 0.9**	**0.2 → 0.5**	**0.5 → 0.9**	**0.2 → 0.5**	**0.5 → 0.9**
1	1	________	No data	No data	No data	↑ 0.94	No data
	2	No data	↓ 0.70	No data	________	________	No data
	3	No data	↓ 0.86	No data	↓ 0.93	________	No data
2	1	↑ 0.44	No data	________	No data	________	No data
	2	________	No data	________	No data	________	No data
	3	________	↓ 0.82	↑ 0.69	No data	________	No data
3	1	________	No data	↑ 0.84	No data	________	No data
	2	No data	↓ 0.74	________	No data	________	No data
	3	No data	↓ 0.71	No data	↓ 0.80	________	No data
4	1	________	No data	↑ 0.83	No data	________	No data
	2	________	↓ 0.59	________	No data	↑ 0.47	No data
	3	No data	↓ 0.41	________	No data	↑ 0.38	No data
5	1	↑ 0.74	No data	↑ 0.75	No data	________	No data
	2	↑ 0.65	No data	________	No data	________	No data
	3	↑ 0.67	No data	________	No data	↑ 0.65	No data

Supplementary Table 3: A strong correlation between the individual amount of PMd-L activation and motion smoothness. For each tested condition and scanning day a correlation coefficient between the individual amount of PMd-L activation and motion smoothness was computed. The analysis was done separately for conditions in which participants shifted from generating non straight (jerky) trajectories to generating straight trajectories (whole-trial fit index = 0.2 → 0.5) and conditions in which participants shifted from generating straight trajectories to generating curved, smooth trajectories (whole-trial fit index = 0.5 → 0.9). The values denote R-square statistic (multiple linear regression using least squares, $p<0.05$). ↑ A positive correlation**.** ↓ A negative correlation**. Underscore** A condition in which no correlation between the two descriptors was found ($p>0.05$). In each condition, the subjects movements'

whole-trial fit indices were either between 0.2 and 0.5 (0.2 → 0.5 group) or between 0.5 and 0.9 (0.5 → 0.9 group) (i.e., one group being void of data). For all conditions in which participants shifted from generating non-straight (jerky) trajectories to generating straight trajectories the correlation between the two descriptors was found to be non-significant or positive whereas for all conditions in which participants shifted from generating straight point-to-point trajectories to generating curved, smooth trajectories the correlation between the two descriptors was found to be non-significant and negative.

Subject #	Day	SEQ		REV		COMP	
		**0.2 → 0.5**	**0.5 → 0.9**	**0.2 → 0.5**	**0.5 → 0.9**	**0.2 → 0.5**	**0.5 → 0.9**
1	1	—	No data	No data	No data	—	No data
	2	No data	↑0.91	No data	—	—	No data
	3	No data	↑0.65	No data	—	—	No data
2	1	—	No data	—	No data	—	No data
	2	—	No data	—	No data	—	No data
	3	—	—	—	No data	—	No data
3	1	—	No data	—	No data	—	No data
	2	No data	↑0.95	—	No data	—	No data
	3	No data	↑0.63	No data	↑0.65	—	No data
4	1	—	No data	—	No data	—	No data
	2	—	↑0.73	—	No data	—	No data
	3	No data	↑0.53	—	No data	—	No data
5	1	—	No data	—	No data	—	No data
	2	—	No data	—	No data	—	No data
	3	—	No data	—	No data	—	No data

Supplementary Table 4: The individual amount of pre-SMA-L activation significantly increases when smoothly concatenating adjacent motion elements. See Supplementary Table 3 for markings. For all conditions in which participants shifted from generating straight point-to-point trajectories to generating curved, smooth trajectories the correlation between the two descriptors was very strong.
